# Detection of bimodal survivin expressions in canine cancer types by flow cytometry compared to immunohistochemistry

**DOI:** 10.3389/fvets.2025.1552415

**Published:** 2025-03-27

**Authors:** Shohei Tsumoto, Kyoichi Tamura, Yuta Nakazawa, Michio Fujita, Kozo Ohkusu-Tsukada

**Affiliations:** ^1^Laboratory of Veterinary Pathology, School of Veterinary Medicine, Nippon Veterinary and Life-Science University (NVLU), Tokyo, Japan; ^2^Laboratory of Veterinary Clinical Pathology, School of Veterinary Medicine, Nippon Veterinary and Life-Science University (NVLU), Tokyo, Japan; ^3^Laboratory of Veterinary Radiology, School of Veterinary Medicine, Nippon Veterinary and Life-Science University (NVLU), Tokyo, Japan

**Keywords:** survivin, flow cytometry, CMM2, CMeC2, LMeC, p815, CT26, B16F10

## Abstract

Animal practice requires both convenience for the owner and risk management for the animal's health. Deterioration due to cancer may associate with poor prognosis under general anesthesia, which need to partial excision for pathological diagnosis. This study aimed to establish rapidly detecting the expression of survivin antigens for cancer vaccines or molecular targeted therapies via flow cytometry (FCM) using the intracellular staining method in tumor samples obtained via needle biopsy without anesthesia. Therefore, survivin expression patterns in each cell lines of canine melanomas, a murine mast cell tumor, a murine colon carcinoma, and a murine melanoma was analyzed by FCM and immunofluorescence microscopy, and compared with immunohistochemical analysis and western blot method. Interestingly, FCM results of the bimodal expression pattern of survivin were suggested to reflect the high fluorescence intensity of its nuclear–cytosol localization and the weak fluorescence intensity of its cytosol alone localization. In a case of canine cancer disease, it was confirmed that survivin expression patterns can be detected via FCM using needle biopsy samples in actual clinical settings. In this study, a novel method via FCM was proposed to quickly determine also survivin localization not only whether the survivin is expressed in cancer cells. The application of cancer vaccine or chemical therapy via this technology can be expected to contribute to improved animal care due to the “one-day first program,” which has been proposed in convenience for owners.

## 1 Introduction

Survivin (BIRC5, API4) is known for its dual biological role in apoptosis inhibition and mitotic progression (proliferative response) in many cancers (1). Five splice variants of survivin have been reported, namely, survivin-2α, survivin-3α, survivin-2B, survivin-3B, and survivin-δ-Ex3, in addition to the survivin wild type (2). These heterogeneous or homogenous dimerization results in the determination of nuclear or cytoplasmic localization and the functions of apoptosis inhibition and mitotic progression (3). Survivin-δ-Ex3 has antiapoptotic functions (inhibits caspase-3) and promotes cell cycle progression via nucleolar localization signals and degradation signals (4). It has also been shown that not only survivin dimers but also survivin monomers participate in regulating apoptosis (5). In highly malignant cancers where the splicing of survivin is progresses, more survivin-δ-Ex3 of five splice variants are formed, which leads to the nuclear localization of the survivin molecule. On the contrary, it was also reported that survivin-2B expression (in cytoplasm) was dominant in benign brain tumors in comparison with the malignant ones (6).

In canine cancer, survivin expression is recognized in various malignant tumors, such as lymphoma (7), malignant melanoma (8), mast cell tumors (9), hemangiosarcomas (10), transitional cell carcinoma (11), osteosarcoma (12), cutaneous squamous cell carcinomas (13), histiocytic sarcoma (14), nasal carcinoma (15), prostatic carcinoma (16), and canine hemangiopericytomas (17). Therefore, the molecular targeted agents using such as 3-cyanopyridine, YM-155, Debio1143, EM1421, LQZ-7I, or TL32711 (18), and immunotherapies as cancer vaccines (19), targeting survivin molecules have been highlighted recently.

In previous cancer treatments for dogs, the pathological diagnosis will take on 5 to 7 days after tumor collection, and surgical treatment will be 1 month later for reasons of hospital's reservation and owner schedule, resulted in tumor growth and metastasis. If a rapid diagnosis of survivin expression can be made today, it will enable measures to be taken immediately to slow the progression of cancer and may contribute to reduce the tumor volume and the metastasis risk before surgical treatment.

In this study, we propose a novel method via FCM to quickly determine whether survivin is expressed in cancer cells. Cancer vaccine or chemotherapy by applying this technology will contribute to also solving problems related to human convenience focused on the field of animal medicine.

## 2 Method

### 2.1 Mice

An intradermal allograft model or xenograft model using murine or canine cell lines was generated by using NOD/SCID mice (female, 8–12 weeks old). All the mice were kept on clean racks under appropriate air conditioning, room temperature, humidity, and a 12-h light cycle in accordance with the ethical guidelines of the Nippon Veterinary and Life Science University.

### 2.2 Cell lines

A total of six cell lines were used: canine malignant melanoma lines [CMM2, CMeC2, LMeC; provided by Dr. Takayuki Nakagawa, Department of Veterinary Surgery, University of Tokyo; (20)], the murine malignant melanoma line B16F10, the murine mast cell tumor line p815, and the murine colon cancer line CT26 (distributed for a fee by the JCRB Cell Bank).

### 2.3 Immunohistochemical analysis (IHC)

After the cell line xenograft or allograft models were prepared, the removed tissue samples were embedded in paraffin blocks, sectioned at 3 μm, deparaffinized in xylene, and rehydrated. Endogenous peroxidase was subsequently treated with 3% H_2_O_2_ solution for 30 min, and antigen retrieval was subsequently performed by autoclaving at 105°C for 25 min (pH 9.0 EDTA). After cooling at room temperature, the samples were washed three times for 3 min with phosphate-buffered saline (PBS). Next, milk blocking was performed, and a rabbit anti-survivin monoclonal antibody (mAb; 1:300, ab134170, Abcam) was applied and incubated overnight at 4°C. After being washed three times for 3 min with PBS, the sections were incubated with an HRP-labeled anti-rabbit secondary antibody (1:300; G0418, Tokyo Chemical Industry, Japan) for 1 h at 37°C. After washing three times for 3 min each, the sections were stained with DAB for 10 min and counterstained with hematoxylin for 1 min.

### 2.4 Western blotting (WB)

WB was performed via standard methods to examine total cellular components and cytosolic fraction components. First, various cell lines were dissolved in a neutral detergent, a portion was centrifuged at 10,000 × g to adjust the total protein amount of the cytosolic fraction, and the total cellular components and cytosolic fraction components were prepared by diluting them in the same ratio. The proteins were separated and subjected to 10% SDS–PAGE. After being transferred to polyvinylidene difluoride (PVDF) membranes treated with 99% methanol, the membranes were incubated with 5% skim milk in PBS-T (10 mM sodium phosphate, 0.15 M NaCl, 0.05% Tween-20, pH 7.5) for 1 h to block non-specific binding. Anti-survivin mAb (1:500, ab134170, Abcam, USA) and anti-β-actin mAb (1:1000, W16197A, BioLegend, USA) were then incubated overnight at 4°C. The membrane was then incubated with an HRP-conjugated anti-rabbit secondary mAb (1:5,000). Survivin or β-actin protein was visualized via an enhanced chemiluminescence (ECL) detection system (GE Healthcare Bio-Sciences). The density of the plot was measured via ImageJ software, and the density ratio of cytosolic survivin was fitted on the basis of total survivin corrected for β-actin density.

### 2.5 Flow cytometry (FCM)

First, the cultured cells or needle biopsy sample cells were treated with 0.25% trypsin-EDTA for 5 min, and the cells were allowed to accumulate. For the intracellular staining method, the cells were fixed with 4% Parafolaldehyde for 60 min. Next, they were reacted with 0.05% Triton-X for 30 min to treat the cell membrane. Finally, they were reacted with an APC-labeled survivin mAb (1:200, ab134170, Abcam) for 30 min and then analyzed via a flow cytometer (Beckman, CytoFLEX).

### 2.6 Immunofluorescence microscopy (IM)

The cells were seeded in 96-well glass-bottom plates (GP96000, Matsunami, Osaka, Japan). Adherent cells were fixed with 4% paraformaldehyde and treated with 0.05% Triton, followed by APC-conjugated survivin mAb (1:200, ab134170, Abcam) treatment. Nuclei were counterstained with DAPI (SeraCare, Milford, IA). Images of the sections were captured via a BZ-X800 fluorescence microscope (Keyence Corporation; Tokyo, Japan) at 400 × magnification to measure the survivin (red) area. The images are displayed in Z-axis slices.

### 2.7 Statistical analysis

The data are expressed as the means ± SEMs. Statistical analysis was performed via one-way ANOVA. A *p* < 0.05 was considered statistically significant.

## 3 Results

### 3.1 Analysis of survivin expression sites by IHC or WB

**Figure 1:**
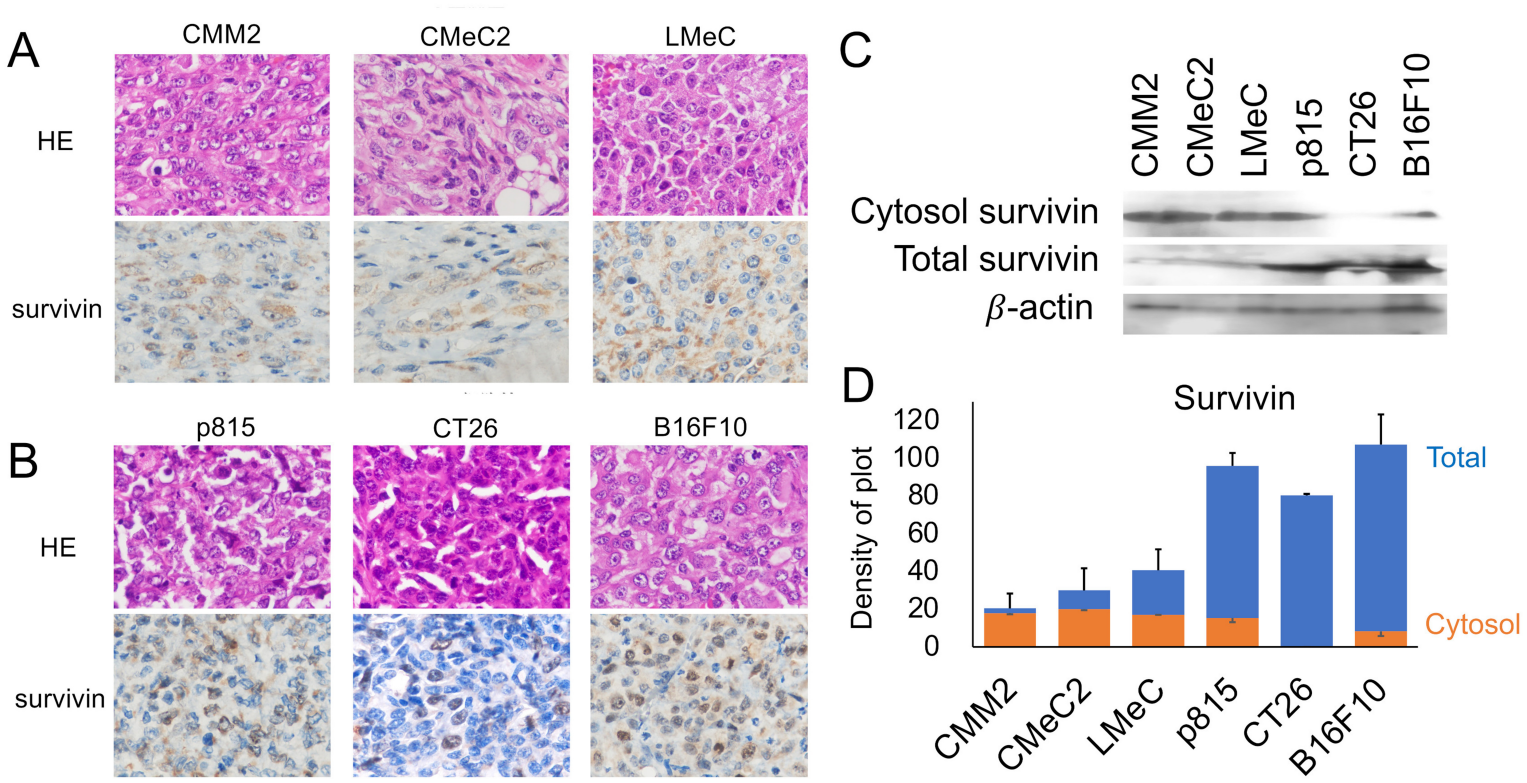
Analysis of survivin expression sites by IHC and WB. **(A)** Images of HE staining and survivin expression via IHC in canine melanoma cell lines (CMM2, CMeC, and LMeC) are shown. **(B)** Images of HE staining and survivin expression via IHC in murine cell lines (p815, CT26, and B16F10) are shown. **(C)** The cytosol and total survivin expression patterns via WB are indicated. **(D)** The expression levels of total survivin (blue bar) and cytosolic survivin (orange bar) corrected on the basis of β-actin expression are shown via density of plot analysis via ImageJ software.

The expression of survivin in the tumor mass from each cell line in the intradermal allograft model or xenograft model in NOD/SCID mice was analyzed via IHC. In canine melanoma cell lines (CMM2, CMeC, LMeC), granular expression of survivin was observed in the cytosol, with little expression in the nucleus. In murine cell lines (p815, CT26, and B16F10), survivin was relatively strongly localized to the nucleus and was expressed in both the cytosol and nucleus in the mast cell tumor line p815 and the melanoma line B16F10. On the other hand, in the colon cancer line CT26, survivin was specifically expressed in the nucleus (Figures 1A, B). The expression of survivin in the total or cytosolic cell fraction was compared with the adjusted total protein amount using β-actin via WB analysis (Figures 1C, D). The amount of total survivin was significantly 2–3 times greater in the murine cell lines than in the canine melanoma cell lines. There were no significant differences between p815 and B16F10 (*p* = 0.447, one-way ANOVA), but CT26 was significantly lower than p815 or B16F10 (*p* < 0.05, one-way ANOVA). The amount of cytosol-localized survivin expression was observed in all the analyzed cell lines except CT26.

### 3.2 Analysis of survivin expression patterns via FCM and IM

**Figure 2:**
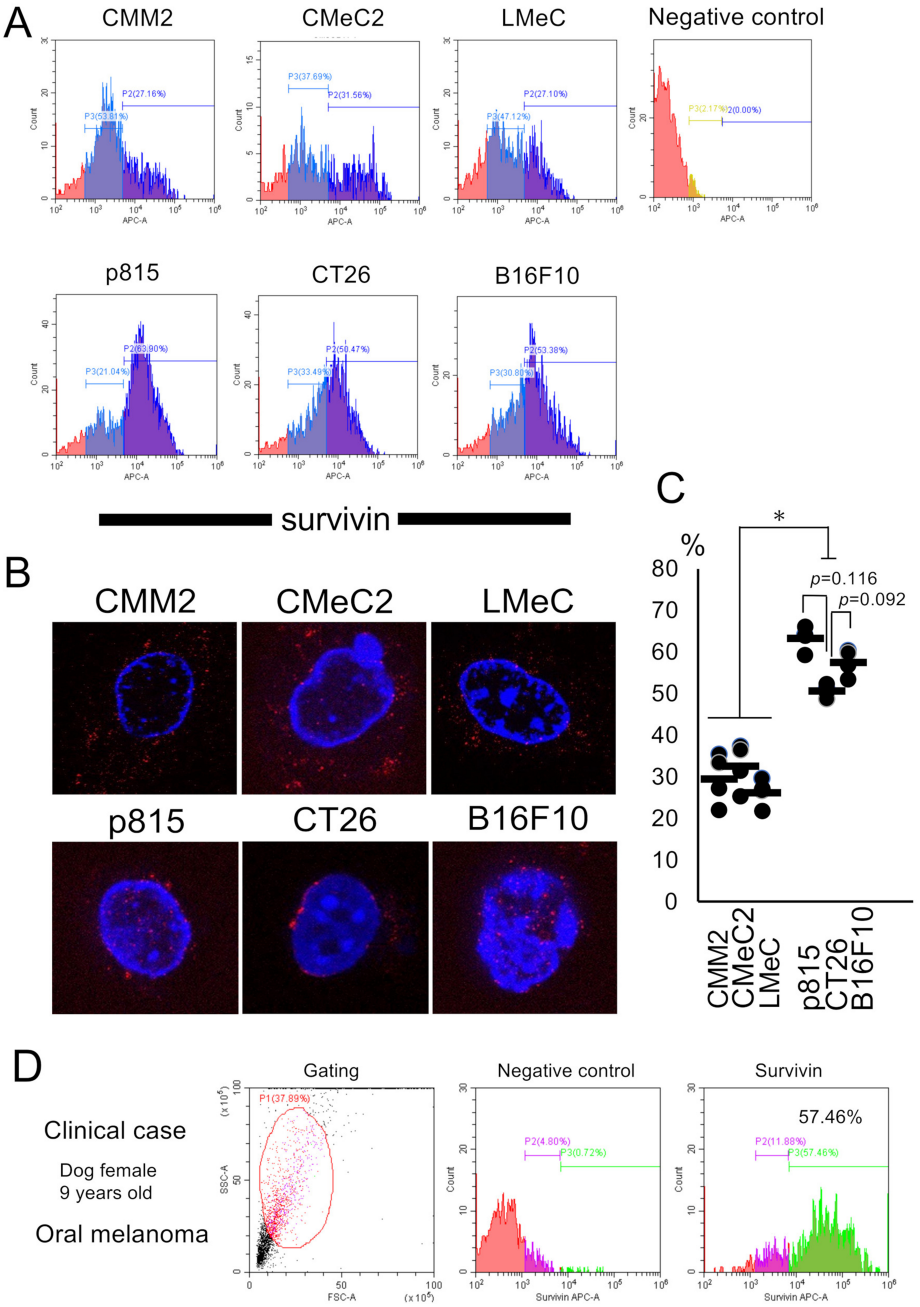
Analysis of survivin expression patterns by FCM and IM and the tests in a clinical case. **(A)** The fluorescence intensity of APC-survivin in each cell line detected by FCM is shown. **(B)** Representative survivin expression patterns in each cell line by IM are shown. **(C)** The percentages of high fluorescence intensity of survivin expression in each cell line are indicated. *p < 0.05, one-way ANOVA. **(D)** Survivin expression after needle biopsy in a clinical case of canine oral melanoma (female, 9 years old) was analyzed via FCM. The percentage of cells with high fluorescence intensity was 57.46%. Similar findings were detected in four other cases of oral melanoma.

The FCM results indicate that the fluorescence intensity of each individual cell is different but that survivin is detected in total cells, including both the cytosol and nucleus. Histogram analysis via FCM revealed that survivin expression was bimodal in each individual cell line. Although the canine melanoma cell lines (CMM2, CMeC2, and LMeC) expressed survivin mainly in the cytosol were few as 27.2, 31.6, and 27.1%, respectively, in percentage of relatively high fluorescence intensities. On the other hand, the murine cell lines (p815, CT26, and B16F10) expressed mainly in nucleus–cytosol or nucleus alone localization were many as 63.9, 50.4, and 53.4%, respectively, in percentage of relatively high fluorescence intensities (Figures 2A, C). The bimodal expression pattern suggested a high fluorescence intensity of nuclear—cytosol localization and a weak fluorescence intensity of cytosol alone localization. The survivin expression in CT26 cells (nuclear alone localization) tended to be lower than that in p815 and B16F10 cells (nuclear—cytosol localization), but the difference was not significant (*p* = 0.116 and *p* = 0.092, one-way ANOVA). The IM results revealed that the expression pattern of survivin in terms of both nuclear localization and cytosolic localization was granular in each individual cell line; however, interestingly, that in CT26 cells was restricted to the nuclear membrane (Figure 2B). These results suggest that survivin expression detected by FCM is not inconsistent with the results of IHC and WB (expression in the cytosol, nucleus, or both in each cell line) and is reasonably consistent.

### 3.3 Survivin expression detected via FCM using needle biopsy samples

A clinical case of canine oral melanoma (female, 9 years old) was evaluated for survivin expression via FCM (Figures 2D). This histogram analysis revealed a bimodal pattern, similar to the FCM analysis using each individual cell line, with high fluorescence expression (57.46%). This survivin expression pattern was similar to that of both the cytosol and nucleus, such as p815 or B16F10 cell line.

### 3.4 “One-day first program” for cancer screening and vaccination

**Figure 3:**
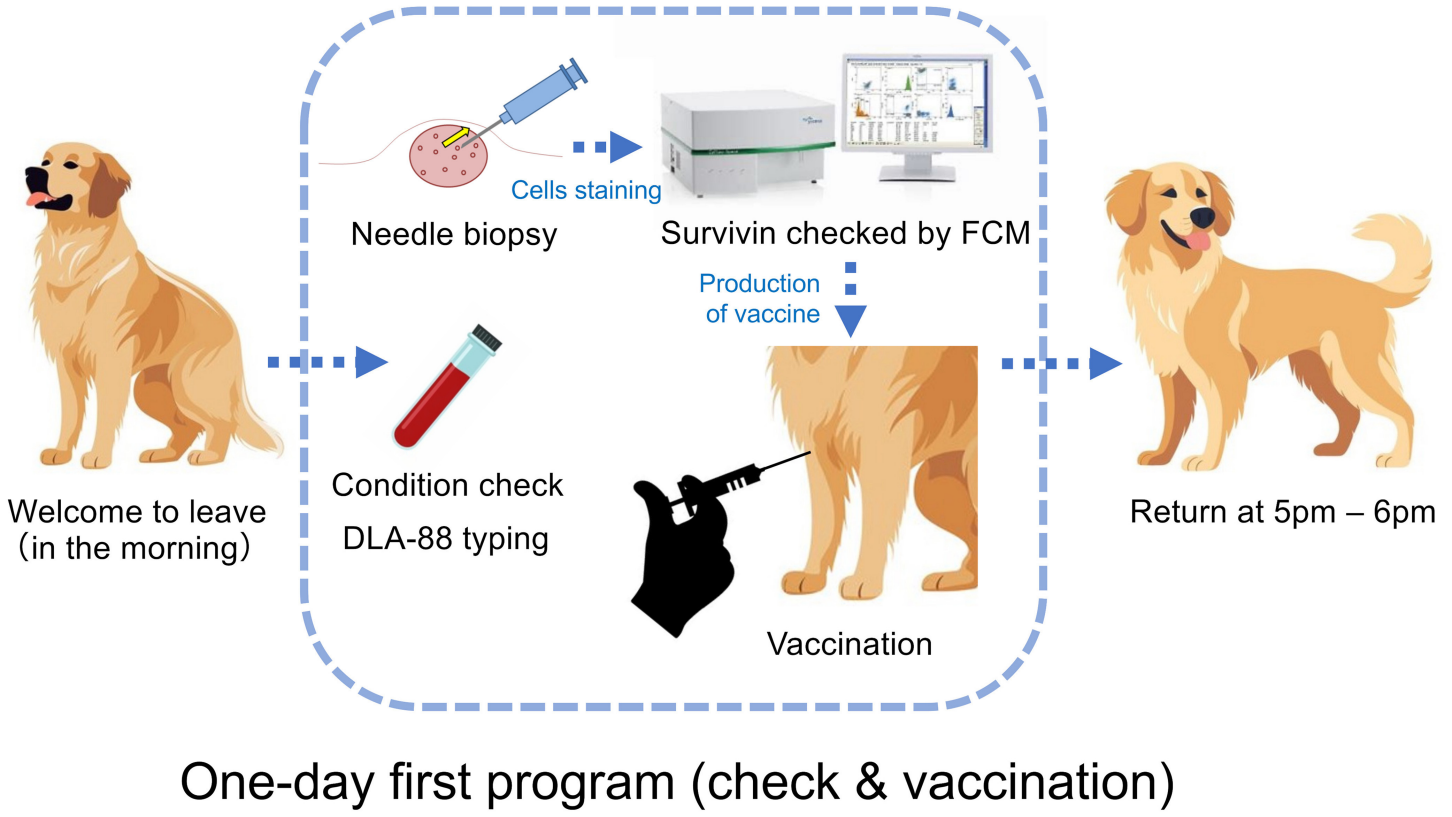
“One-day first program” for a cancer check and vaccination. At the university's veterinary hospital, after an interview and an informed consent form (ICF) with the owner in the morning, the patient dog is taken in, and a general CBC blood test is performed to check its health condition. Needle biopsy will be performed on the tumor, the cells will be stained via an APC-conjugated anti-survivin mAb after detergent treatment following cell membrane fixation, and the presence or absence of survivin expression will be examined by FCM. If the tumor expresses survivin, the vaccine after production will be administered intradermally, such as through the axilla. After confirming that there is no anaphylactic reaction, the patient's dog waits until the owner returns to pick up after 5 p.m.

A cancer screening and therapy program that is highly convenient for dog owners is important. The “one-day first program” proposed here is possible to be implemented immediately by avoiding the owner's schedule issues (Figure 3).

The pathological diagnosis will take on 5 to 7 days after tumor collection, and surgical treatment will be performed 1 month later for reasons of hospital's reservation and owner schedule. During this time, the tumor volume increases ~4fold, and both the difficulty of surgery and the risk of metastasis increase. The “one-day first program” as therapy strategy that utilizes and applies the technology established in this study, whose introduction will contribute to simplifying surgical therapy 1 month later by reducing the tumor volume and the risk of metastasis. Thus, depending on the various cases, cancer vaccines using this program can be used not only for (1) the purpose of preventing recurrence but also for (2) active treatment purposes, such as achieving complete or partial remission, and for (3) the purpose of improving poor prognosis when recurrence has occurred after surgery, chemotherapy, radiation therapy, etc., and retreatment is difficult.

## 4 Discussion

A 2006 study of canine mast cell tumors reported no association between the nuclear or cytosol localization of survivin and prognostic survival rates (9). In a recent study of canine hemangiopericytomas, nuclear survivin expression was observed in all 41 cases (100%), but the proportion of nuclear survivin-expressing cells in the entire tumor mass ranged from 1 to 12%. In contrast, cytoplasmic survivin expression was observed in 31/41 cases (76%), and the proportion of cytoplasmic survivin-expressing cells in the entire tumor mass was 75% or greater. However, the important point of this report is that a statistical association was demonstrated in which every 1% increase in nuclear survivin in the tumor mass was associated with a 1.15-fold increase in the risk of immediate mortality (17). Future research should focus not only on the presence or absence of survivin expression for each type of cancer but also on the localization of survivin in the nucleus and cytoplasm to verify the prognosis.

In this study, the bimodal expression pattern of survivin was suggested to reflect the high fluorescence intensity of its nuclear–cytosol localization and the weak fluorescence intensity of its cytosolic alone localization resulting from FCM in various cell lines. This makes it possible to use FCM technology on clinical specimens obtained via needle biopsy to determine whether the survivin of cancer cells is localized in the cytoplasm alone or both nucleus and cytoplasm. Previous reports (6) have shown that of the five splice variants of survivin, survivin-δ-Ex3 (expressed in the nucleus) is expressed in highly malignant tumors, whereas survivin-2B (expressed in the cytosol) tends to be expressed in relatively benign tumors. In the future, when fluorescent-labeled anti-survivin-2B mAbs and anti-survivin-δ-Ex3 mAbs become available to canine tumor cells, the test via FCM may be able to reflect the prognostic status.

## 5 Conclusions

In this study, a novel method via FCM was proposed to quickly determine also survivin localization not only whether the survivin is expressed in cancer cells. The application of cancer vaccine or chemotherapy via this technology can be expected to contribute to improved animal care due to the “one-day first program.” This technology is also expected to be applicable to other proteins involved in cancer progression.

## Data Availability

The datasets presented in this study can be found in online repositories. The names of the repository/repositories and accession number(s) can be found in the article/Supplementary material.
