# Supplementary material for: Detection of bimodal survivin expressions in canine cancer types by flow cytometry compared to immunohistochemistry
Source: Front Vet Sci. 2025 Mar 27;12:1552415. doi: 10.3389/fvets.2025.1552415 (PMC11984292; doi:10.3389/fvets.2025.1552415)
Supplement: Supplementary file 1 [file Data_Sheet_1.pdf]

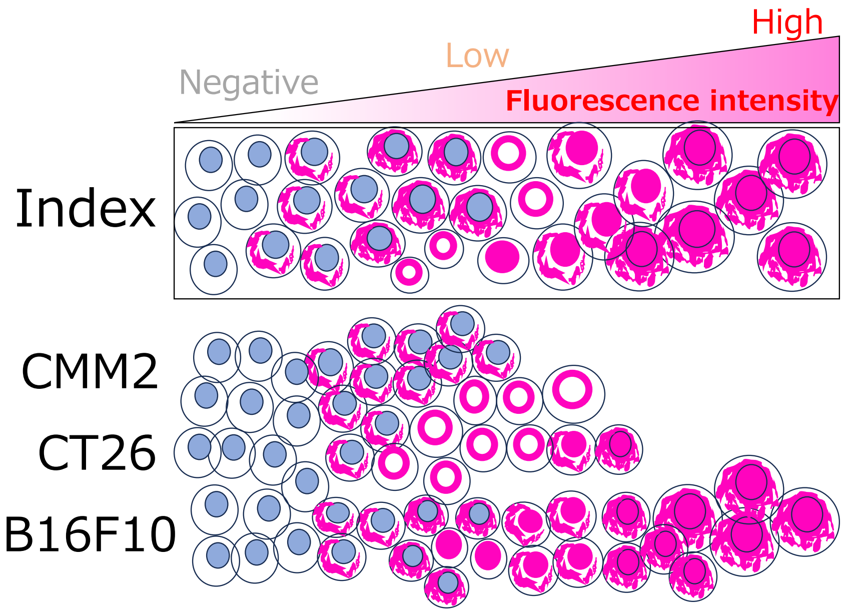

Figure S1. Aggregation of multiple survivin expression patterns

It is predicted that multiple survivin expression patterns exist heterogeneously in a single cell line. The Index shows that the fluorescence intensity increases in the following order: cells expressing in the cytosol, cells expressing in the nucleus, and cells expressing in both the nucleus and the cytosol. For example, in CMM2, it is expected that most cells will be composed of cells with survivin expression in the cytosol, in CT26, most cells will be composed of cells with survivin expression on the nuclear membrane, and in B16, most cells will be composed of cells with survivin expression in both the cytosol and nucleus.
